# Diffusion of Valley-Coherent Dark Excitons in a Large-Angle Incommensurate Moiré Homobilayer

**DOI:** 10.1021/acs.nanolett.5c00456

**Published:** 2025-03-14

**Authors:** Arnab Barman Ray, Trevor Ollis, K.R. Sethuraj, Anthony Nickolas Vamivakas

**Affiliations:** † The Institute of Optics, University of Rochester, 480 Intercampus Dr, Rochester, New York 14627, United States; ‡ Department of Physics and Astronomy, University of Rochester, Rochester, New York 14627, United States; ¶ Center for coherence and quantum optics, Department of Physics, University of Rochester, 480 Intercampus Dr, Rochester, New York 14627, United States; § Materials Science, University of Rochester, Rochester, New York 14627, United States

**Keywords:** 2D materials, Moiré superlattice, Intralayer excitons, Optoelectronics

## Abstract

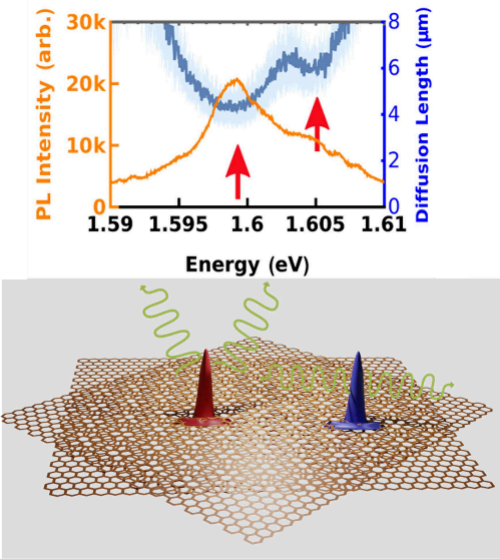

Recent research in twistronics, particularly in small-angle twisted bilayers of transition metal dichalcogenides, has uncovered exciting phenomena like periodic arrays of excitonic quantum emitters, exotic many-body states, and long-lived interlayer excitons. However, less explored has been the physics of large-angle, incommensurate bilayers, where periodicity breaks down. In this study, we demonstrate the emergence of a brightened dark intralayer exciton in a twisted n-doped molybdenum diselenide homobilayer. This dark exciton diffuses more efficiently than bright excitons or trions, with diffusion lengths over 4 μm. Temperature-dependent spectra show a brightened dark trion, and we observe a robust valley coherence. This unique behavior is attributed to a small mixing of spin-resolved conduction bands, caused by a lack of out-of-plane reflection symmetry and strong dielectric contrast. Our findings open new possibilities for valleytronic devices using valley-robust “mixed” dark excitons.

Moiré hetero- and homobilayers of transition metal dichalcogenides(TMDCs) have been shown to host correlated electronic phenomena^1−8^ and arrays of programmable quantum emitters.^9−17^ Large-angle twisted homobilayers (10° < θ < 50°) without a periodic superlattice have been less explored. While the Moiré superlattice is active only at small angles^18^ and allows for the trapping of excitons, at high angles, this periodicity is broken. The condition for commensurability or periodicity in a twisted bilayer system of two honeycomb lattices is provided by the equation^19^
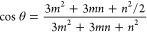
where θ is the twist angle and *m* and *n* are a pair of coprime positive integers. Immediately, it can be discerned that angles where the value of the cosine is irrational do not exhibit periodicity. However, given that there are an infinite number of coprime pairs of positive integers, it is possible to have a rational number expressed in the form of the previous equation arbitrarily close to the irrational number in question. This leads to a highly sensitive, if not wholly chaotic period at large angles.^20^ With the limitations of current fabrication techniques,^21^ this leads to an essentially aperiodic structure without the band modulating and flattening effects that a Moiré superlattice usually endows.

Without an active and periodic Moiré superlattice, these large-angle bilayers may seem to hold little promise in terms of the originally intended applications in quantum computation for these systems. A high twist angle pushes diffusive interlayer excitons^22^ out of the light cone while limiting the possibility of simulating correlated states in periodic lattices^23^ due to an effective uncoupling of electronic communication between the two layers, as is seen in graphene. However, as we show in this work, this class of aperiodic bilayers can be interesting in its own right. We focus on a large-angle twisted homobilayer of n-doped molybdenum diselenide. These systems have been explored in a recent work^24^ over a range of twist angles. We show, using standard microphotoluminescence (PL) experiments, that at large twist angles, the proximity of a highly polarizable monolayer to the other alters its optical properties. As previously reported,^24^ we observe a large redshift of the trionic and excitonic resonances in the bilayer. This redshift can be partly attributed to the large polarizability of the proximal monolayer, similar to the much smaller redshift experienced by a free-standing monolayer when encapsulated by a high dielectric constant insulator such as hBN.^25^ Thus, the large-angle twist of the bilayer serves to prevent the heterostructure from becoming an indirect bandgap semiconductor which is the case for a Bernal-stacked (0°) bilayer,^26^ while at the same time having the Moiré superlattice potential inactive.

Upon investigating the spatial diffusion of bound complexes under steady-state continuous-wave (CW) excitation at low intensities across the PL spectrum, we uncover quenching of diffusion lengths at energies of high quantum yield or maximum PL intensity. Investigating diffusion lengths points us toward the presence of a more diffusive bound species at a slightly higher emission energy than the bright spin-singlet exciton. By carefully deconstructing the spectra, we discover that this new species of exciton diffuses more efficiently than the bright excitons or trions. Investigating the temperature dependence of the PL reveals evidence for a population transfer to this brightened dark exciton. Population transfer to higher-energy dark excitonic states is responsible for the decrease in quantum yield with increasing temperature in single monolayers of MoSe_2_ and MoS_2_ with the point group symmetry *D*_3*h*_. It is well documented and understood.^27−29^ However, we find that for a large-angle twisted bilayer this population transfer still allows us to capture some of the PL emitted from this spin-forbidden dark exciton, as a result of the removal of the out-of-plane reflection symmetry, causing the point group of both monolayers to reduce to *D*_3*v*_. The emission resulting from these dark excitons is usually suppressed due to the much smaller radiative rate that accompanies spin-flip electronic transitions in monolayers.^30^ This brightening of this triplet trion is similar to what has been predicted and observed for the case of interlayer excitons in WSe_2_–MoSe_2_ heterobilayers.^31,32^ Furthermore, we show that these dark triplet excitons are more diffusive than their bright counterparts, with diffusion lengths exceeding 4 μm. Dark excitons are usually long-lived and optically decoupled from the environment, and serve as a reservoir for their bright counterparts, playing a crucial role in the condensation of excitons in other well-studied semiconductor platforms.^33,34^ Hence, it is important to understand their properties.

The monolayers and hBN (high-pressure anvil growth) were mechanically exfoliated from high-quality bulk samples obtained from 2D Semiconductors. The individual flakes were then assembled step-by-step under an optical microscope using dome-shaped windows constructed from cured PDMS with a thin pane of PPC (poly propylene carbonate). After the device was constructed the assembly was heated to release it when in contact with the DBR chip (with an additional 98 nm of SiO_2_ on top). The SiN-terminated distributed Bragg reflector was fabricated using a PECVD method, with 10.5 pairs of SiN/SiO_2_. The second sample was fabricated similarly, with a tear-and-stack technique on an SiO_2_/Si substrate.

The measurements were carried out using a custom-made confocal microscope. A 532 nm DPSS laser is focused into a submicrometer-diameter spot using a 0.70 NA objective lens in a closed-cycle cryostat (Montana Instruments) at 6 K. The emission spot is relayed through the objective and imaged onto the CCD camera using an achromatic lens system. One of the achromatic lenses is stepped longitudinally to minimize the effects of longitudinal color in the system between the measurements of the laser spot and the photoluminescence as they differ in their wavelengths considerably. The collected PL is then analyzed using a Princeton Instruments spectrometer (Acton SP-2750i) and an LN2 cooled Pylon CCD camera. The cylindrical symmetry of the measuring apparatus allows us to project the data along one Cartesian axis parallel to the spectrometer grating. An Msquared Sprite XT femtosecond pulsed ti-saph (80 MHz repetition rate) is used at 790.5 nm for the second harmonic generation measurements. A Coherent Chamaeleon II laser is used in its continuous-wave operation (alignment) mode at 735 nm for some of the measurements. Measurements on the second sample were performed in an Attodry 1000 magneto-optics setup with a 0.82 NA objective and a home-built polarization-sensitive microscope.

Figure 1(a) provides an optical micrograph of the homobilayer investigated in this work. We mechanically exfoliate and assemble monolayers of n-type MoSe_2_ (2D semiconductors) and thin, flat flakes of hBN (2D semiconductors) on a distributed Bragg reflector (see methods in the Supporting Information) with its reflection band centered at 770 nm to optimize the PL signal collection. Using a pulsed Ti-sapphire laser at 790.5 nm, we collect the copolarized second harmonic signal generated from the more accessible bottom monolayer and the homobilayer as a function of the laser polarization angle. After accounting for the effects of the beamsplitters in the signal and collection path, the corrected SHG signal is presented in Figure 1(b) with their respective fits, revealing the twist angle to be 41.27 ± 1.55°. We note that the collected SHG signal also validates the high quality of the fabricated sample and that there is minimal strain present^35,36^ away from the visible bubbles in the micrograph. We probed the photoluminescence spectra in a confocal microscopy setup (0.70 NA microscope objective) where the sample was cooled to cryogenic temperatures (all measurements are at 6 K unless otherwise specified). Figure 1(c) shows the PL signal from the monolayer and bilayer. We observe a large redshift of the trionic and excitonic resonances of Δ_*T*_ = 30.4 meV and Δ_*X*_ = 32.8 meV, due to enhanced dielectric screening.

**Figure 1:**
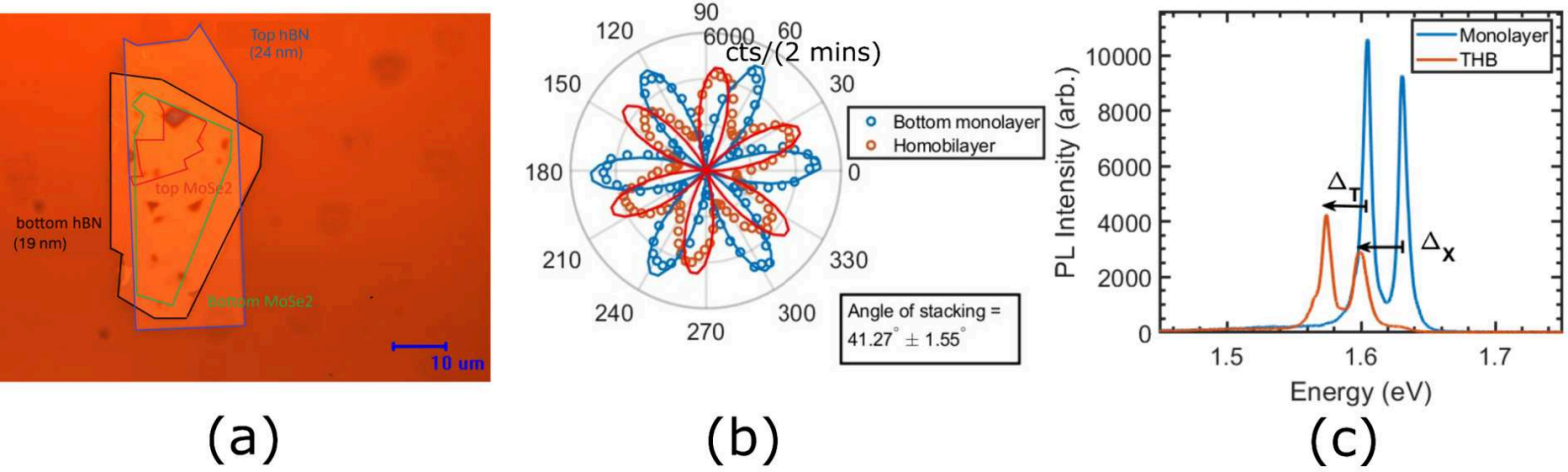
(a) Optical micrograph of the homobilayer, (b) copolarized second harmonic signal from the bottom monolayer and the bilayer used to estimate the twist angle, (c) PL spectrum of the monolayer and bilayer with the shifted trionic (T) and excitonic (X) peaks at 4K, 532 nm CW excitation.

Next, we shift our attention to the diffusion of the excitonic and trionic complexes across the emission spot in both the monolayer and the bilayer under steady-state excitation. While it has been demonstrated that at small angles the localizing effects of the Moiré potential impedes the diffusion for interlayer and intralayer excitons,^37−39^ the case for large-angle bilayers is less explored either theoretically or experimentally. We focus on the diffusion lengths obtained for different species in the monolayer and bilayer. We note that further experimental work involving measurements of PL lifetime would help calculate the diffusion coefficients. However, the focus of this work is to chronicle how diffusion lengths were used to identify the brightened spin-forbidden dark exciton.

Upon excitation with a power of 10 μW in a spot of diameter ∼1 μm, we image the emission spot on the CCD camera of our spectrometer setup and record the spatio-spectrum of the monolayer and the bilayer in Figure 2(a) and (d). Under steady-state excitation, neglecting the effect of exciton–exciton interactions, we fit the spatial extent of the PL intensity as a function of emission energy using the diffusion equation
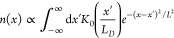
where *K*_0_ is the modified Bessel function of the second kind.^37,40,41^ We determine the laser spot line width *L* by fitting it with a Gaussian function. This fit provides us with the diffusion lengths *L*_*D*_ as a function of emission energy. We verify that the diffusion lengths thus obtained do not change considerably over 3 orders of magnitude of the excitation intensity in the Supporting Information (see Figure S1). Note that the diffusion equation is less appropriate for modeling trion diffusion due to local electric field effects from donor atoms which can modify their dynamics.^42^ However, the quenching of the diffusion lengths for trions at energies corresponding to high quantum yield (high PL intensity) denoted by red arrows in Figure 2(b), (c), (e) and (f) highlights the accuracy of our measurements. Moreover, our data captures the fact that trions diffuse less as compared to excitons due to their larger effective mass^43^ and the aforementioned effects which is well documented in the literature.^42,44^ Figure 2(b), (c), (e) and (f) show that the spectral variation of the diffusion lengths qualitatively resembles a reflection of the PL spectra about the horizontal axis.

**Figure 2:**
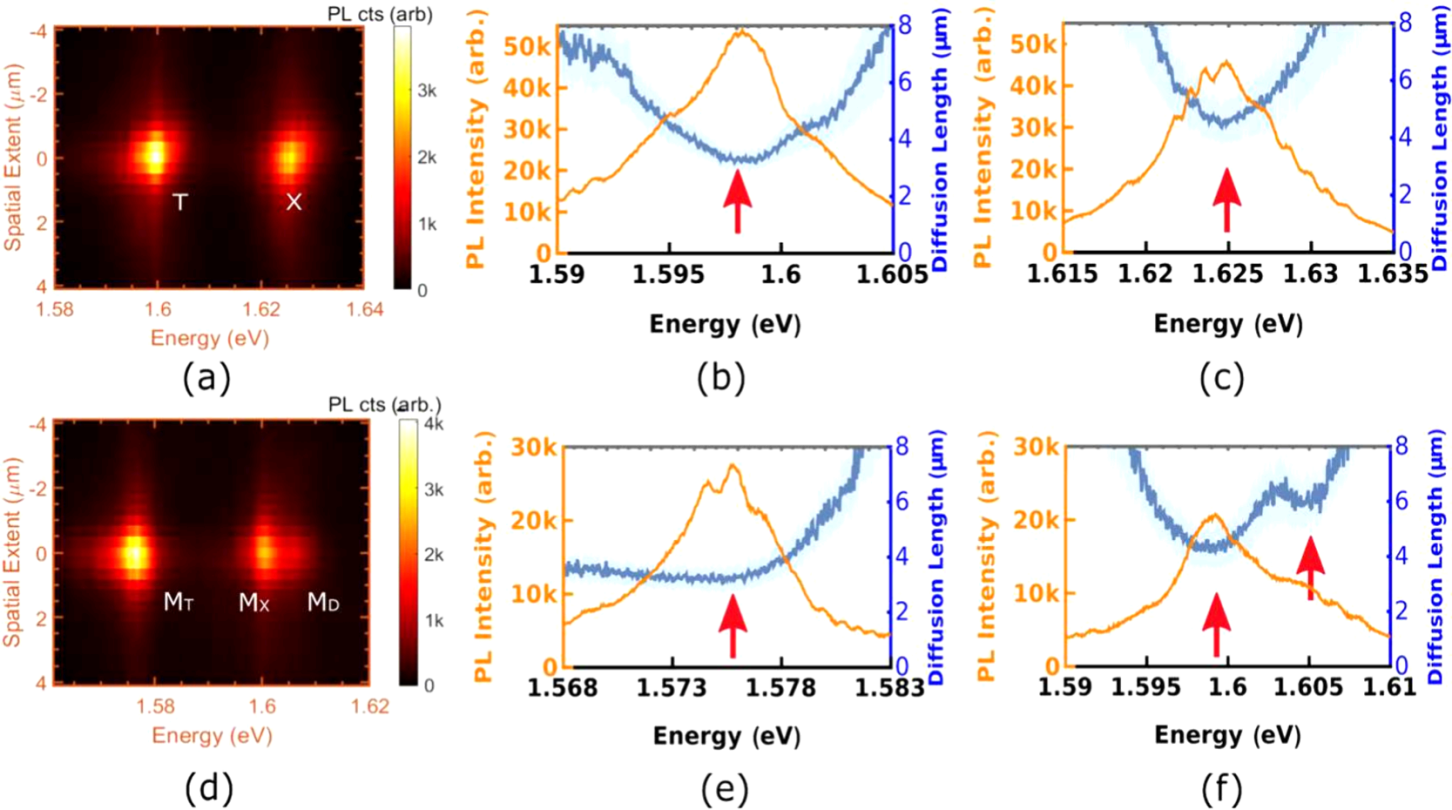
(a) Spatio-spectrum of the monolayer, PL spectrum and the spectral variation of diffusion lengths of (b) the trion (T), and (c) the exciton (X). (d) Spatio-spectrum of the homobilayer and the spectral variation of diffusion lengths of (e) the trion (M_T_), and (f) the exciton (M_X_). Deep blue lines trace the obtained diffusion lengths while light blue shaded regions demarcate the 95% confidence intervals from the fits.

For any diffusive bound complex, the rate of population decay is given as a sum of the radiative and nonradiative rates, Γ_*tot*_ = Γ_*r*_ + Γ_*nr*_. The PL lifetime is given as , where τ_*r*_ = Γ_*r*_^–1^ and τ_*nr*_ = Γ_*nr*_^–1^. As the diffusion lengths theoretically are given by  (where *D* is the diffusion coefficient), substituting this gives, . Using the relation satisfied by the intrinsic PL quantum yield given as , it is straightforward to arrive at the equation, . This relation explains the quenching of the diffusion lengths at energies of high PL intensity or quantum yield.

Figure 2(d) and (f) hints at the presence of a less bright species of exciton (which we label M_D_) at a slightly higher energy than the bright exciton M_X_. At this point, it is impossible to ascertain whether this species is fundamentally different from the bright exciton or whether it is a result of inhomogeneity or dielectric disorder^44^ in the sample. Moreover, while Figure 2(e) and (f) seem to indicate that both M_X_ and M_D_ diffuse much more efficiently than compared to M_T_ or even the monolayer exciton X, the spectral proximity of these two species may cause leakage of the tails of their respective spectra at the peak energies of each other, and thus artificially inflating their actual diffusion lengths. To circumvent this problem, we try to disentangle the contributions of each of these two species. The spectral slices that make up the spatio-spectrum lend themselves well to double-Lorentzian fits (see Figure S2 in the Supporting Information). We are thus able to investigate the diffusion lengths of both of these species separately in Figure 3.

**Figure 3:**
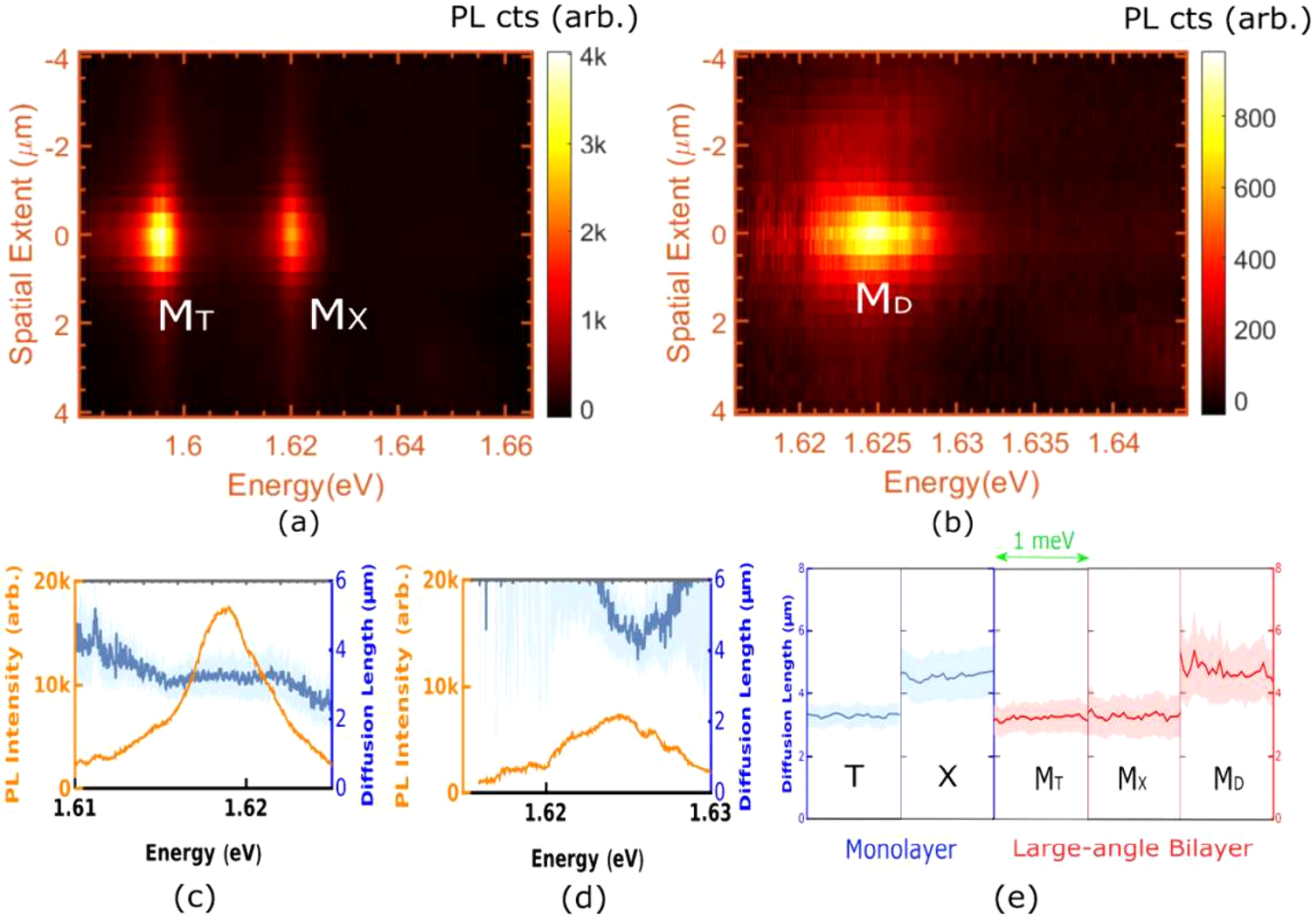
(a) Corrected spatio-spectrum of the bilayer trion and bright exciton and (b) corrected spatio-spectrum of the bilayer dark exciton, (c) spectral variation of diffusion length for the bright exciton, (d) spectral variation of diffusion length for the dark exciton. Deep blue lines trace the obtained diffusion lengths while light blue shaded regions demarcate the 95% confidence intervals from the fits. (e) Diffusion lengths of different species in the monolayer and bilayer over a spectral width of 1 meV across their respective peak PL intensities. Deep blue (red) lines trace the obtained diffusion lengths while light blue (red) shaded regions demarcate the 95% confidence intervals from the fits for the monolayer (bilayer).

We next compare and contrast the diffusion lengths of different species in Figure 3(e). For the monolayer, the exciton diffuses more efficiently than the trions, which face an inward electrostatic force from donor atoms, altering their diffusion significantly. For the bilayer, our results indicate that despite the absence of a periodic Moiré superlattice, the diffusion of bright excitons is suppressed. We suggest that the suppression of excitonic diffusion in these bilayers may arise from induced dipole interactions between the bright excitons and the donor atoms across both monolayers, leading to a qualitatively different behavior than the bright excitons in monolayers. We note that the less bright excitonic species M_D_ diffuses more efficiently as compared to the bright excitons, which may arise from a relatively longer lifetime.^45,46^

**Figure 4:**
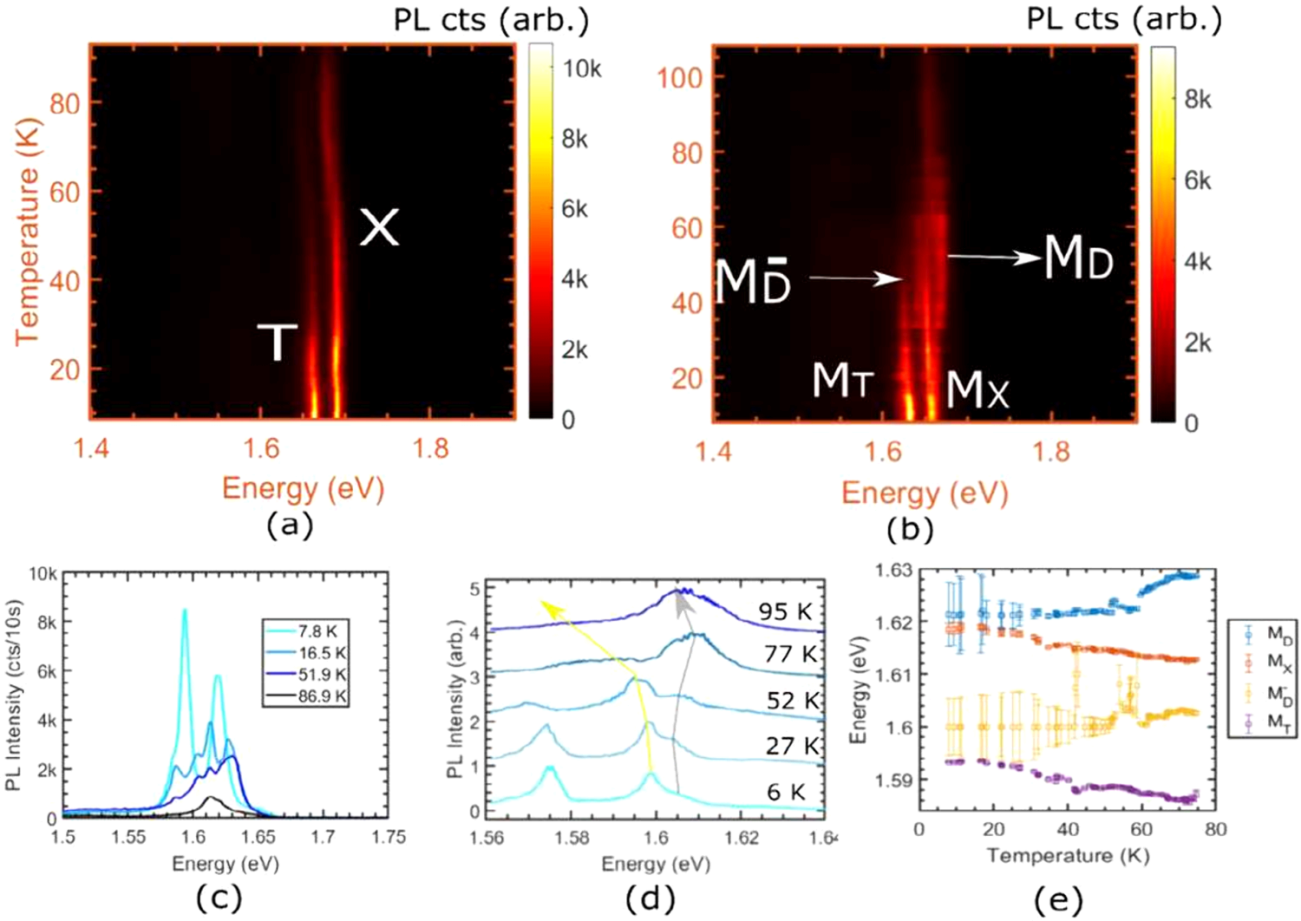
Evolution of PL with temperature for the (a) monolayer and (b) bilayer. (c) PL spectra at different temperatures exhibitng four separate peaks. (d) Dependence of peak energies of bright exciton (yellow arrow) and dark exciton (dark arrow) with temperature. (e) Extracted peak energies as a function of temperature.

To determine the nature of the more diffusive species, we trace the PL signal as a function of the sample temperature. The evolution of diffusion lengths with temperature is provided in the Supporting Information. Figure 5(a) exhibits the temperature dependence of the PL from the monolayer. We notice the monotonic redshift with increasing temperature^47^ and the decrease in PL yield. This is due to the presence of higher energy dark states in MoSe_2_. This decrease in quantum yield is opposite to that of tungsten-based TMDC monolayers, where the presence of low-lying dark states leads to an increase in PL yield with increasing temperature. We trace the PL from the bilayer in Figure 4(b). We note evidence for a visible population transfer to the now-brightened dark states at around 30 K, which corresponds to a thermal energy of ∼2.5 meV, about half of the difference in the peak energies of M_X_ and M_D_. Around that temperature, we detect evidence of a brightened dark trionic state M_D_^–^^48^ (see Figure S5 in the Supporting Information). The extra binding energy of the dark trion at 30 K is 24 meV and is close to the binding energy of the bright trion (27 meV) at the same temperature. The four excitonic and trionic species in question are clearly identifiable in the spectra as four separate peaks at 16.5 K in Figure 4(c). The dark species investigated in this work are intravalley direct dark excitons and trions, and the PL emission is not phonon-assisted, which can be surmised from the relative positions of their peak energies from that of their bright counterparts. Finally, we report the unusual, nonmonotonic behavior of the energy of the dark exciton in Figure 4(d) and (e). In contrast to the continuous redshift of the bright exciton with temperature, the dark exciton initially undergoes a considerable blue shift in its energy before it starts to redshift. This, too, points to a different band origin of the electron in the dark exciton. The cause for this behavior may be analogous to the anti-funneling effects observed for momentum-forbidden dark excitons^49^ in single tungsten-based monolayers, which also arises from a difference in how the electron bands evolve under strain, or in this case, temperature.

**Figure 5:**
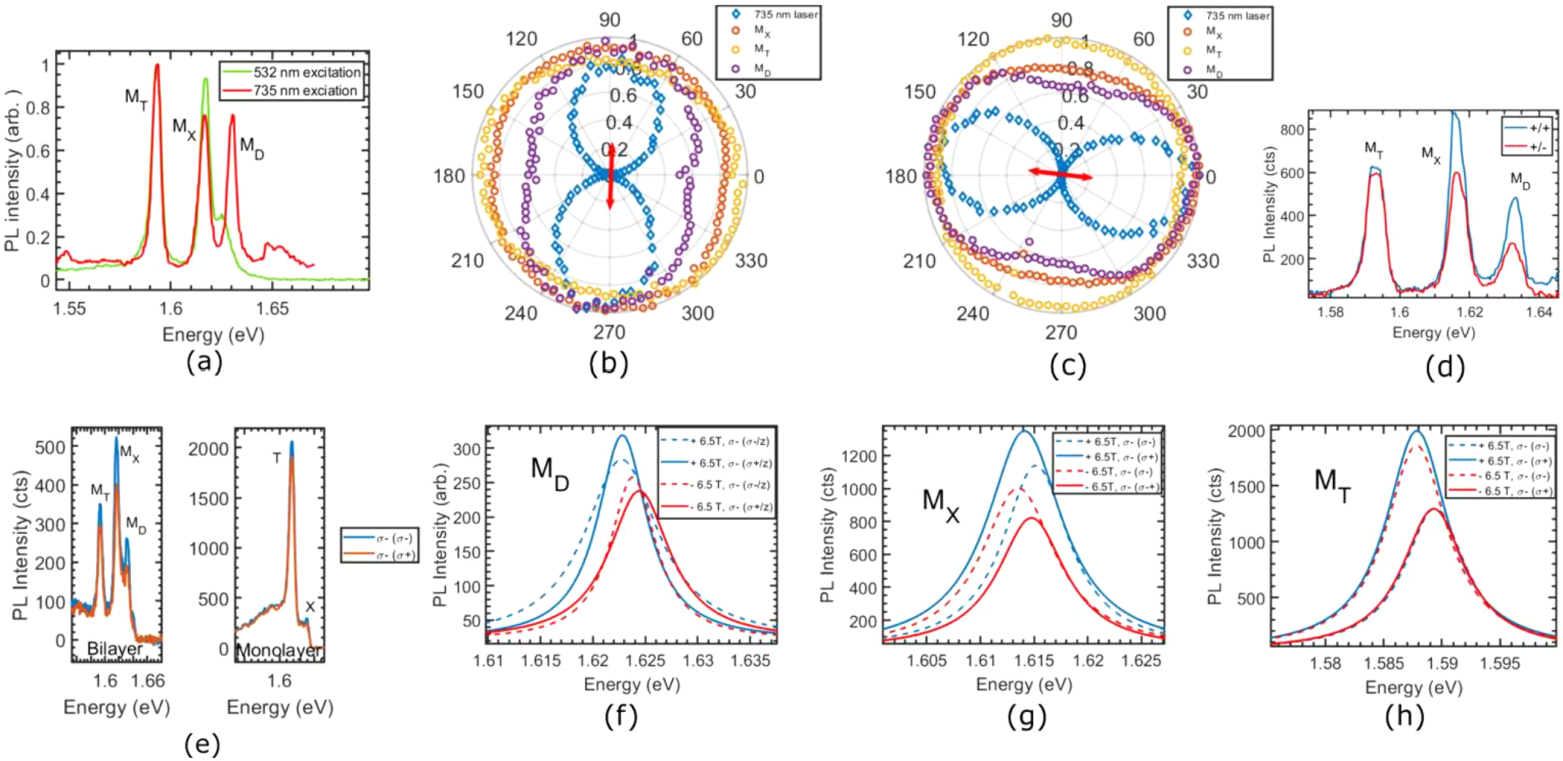
(a) PL spectrum of the bilayer at different excitation photon energies, (b) and (c) integrated PL intensities of the three species (from Lorentizan fits) and the laser, as a function of detection polarization angle at an excitation power of 28 μW, (d) PL spectrum of bilayer for co- and cross-polarizations with respect to the linearly polarized laser, (e) net valley polarization of the bilayer (left) and monolayer (right) with 735 nm excitation at zero magnetic field, (f), (g), and (h) separate fits of PL spectra for the three species (dark exciton, bright exciton and bright trion respectively) with circular polarization selection and magnetic field, with 532 nm excitation at 50 μW. Sample temperature was kept at 12.5 K for all these measurements.

Finally, we study the polarization-resolved properties of the observed bound complexes in a second sample (40° ± 2°, tear-and-stack) with a high-NA (0.82) confocal microscope. We find that using a near-resonant laser energy leads to better-resolved PL spectra and preferential formation of the dark exciton in Figure 5(a). By investigating the quantum valley coherence of the three species, we found, to our surprise, that the dark exciton exhibits an improved and robust valley coherence as opposed to the other excitonic resonances in Figure 5(b), (c) and (d). The species also demonstrate an appreciable amount of valley polarization with 735 nm excitation with no magnetic field (Figure 5(e)). The valley coherence substantiates the claim that the M_D_ emission does not arise from a disorder/defect as these emitters are usually linearly polarized and do not follow the excitation laser polarization.^50^

The data are surprising for two reasons - the first being that MoSe_2_ monolayer is exceptional among its family of TMDCs in that excitons, while bright at cryogenic temperature, do not possess any appreciable valley polarization (or coherence) with nonresonant or near-resonant excitation.^51^ Several reasons have been suggested for this in the literature, ranging from D’yakanov-Perel’, Elliott-Yafet, and MSS mechanisms,^51−53^ as well as a resonance of an optical phonon mode with the conduction-band spin splitting.^54^ Second, the emission from a dark exciton in MoSe_2_ monolayer is originally z-polarized. While collection by a high-NA infinity-corrected objective is possible, this should convert the z-polarization to a radially polarized beam^55^ which should be insensitive to selection by a quarter-wave plate/linear-polarizer combination, especially to a bucket detector such as our fiber-spectrometer-CCD combination. Hence, we investigate the valley splitting of the species with a magnetic field in a Faraday configuration. We find, in Figure 5(f), (g), and (h), that in contrast to the bright species, the dark exciton displays an equivalent energy shift for emission of both handedness under excitation of a single valley with circularly polarized light. This indicates that the majority of the PL emission is from a single valley, while confirming that a substantial portion of the PL collected is primarily z-polarized (radially polarized). Furthermore, the presence of a nonzero valley polarization and the subsequent valley coherence indicates that the emission from dark exciton is not purely z-polarized and hints at a spin-mixing of the two conduction bands due to the broken symmetry of the bilayer. These ”mixed” dark excitons seem to be comparatively well-shielded from the intervalley scattering processes that plague the bright excitons in both the bilayer and the monolayer.

To summarize, we uncover the brightening of the spin-forbidden dark exciton and dark trion in a large-angle incommensurate Moiré homobilayer. We identify a more diffusive species by analyzing the spectral variation of diffusion lengths in the PL spectrum, which we assign to the dark exciton. Investigating the temperature dependence of the PL spectrum leads us to discover the population transfer effects that are otherwise undetectable in the monolayer. The study of steady-state spatial decay of the valley coherence is an interesting area of future work. We discover that these dark excitons are slightly mixed and display a degree of valley addressability which is more robust than its bright counterparts. Diffusive, robust, valley-addressable dark excitons may pave the way for future valleytronic devices. Our results uncover several interesting facets of exciton photophysics in these less-explored large-angle bilayer systems.

## Supporting Information Available

More data and analysis complementing the results presented in the main paper (PDF)
