# Supplementary material for: Diffusion of Valley-Coherent Dark Excitons in a Large-Angle Incommensurate Moiré Homobilayer
Source: Nano Lett. 2025 Mar 14;25(12):4995–5002. doi: 10.1021/acs.nanolett.5c00456 (PMC11951149; doi:10.1021/acs.nanolett.5c00456)
Supplement: Supplementary file 1 — nl5c00456_si_001.pdf [file nl5c00456_si_001.pdf]

Supporting Information for 'Diffusion of valley-coherent dark excitons in a large-angle incommensurate Moiré homobilayer'

Arnab Barman Ray,[†] Trevor Ollis,[‡] Sethuraj K. R.,[†,¶] and Anthony Nickolas Vamivakas[†,¶,§,‡]

†*The Institute of Optics, University of Rochester, 480 Intercampus Dr, Rochester, NY 14627, USA*

‡*Department of Physics and Astronomy, University of Rochester, Rochester, NY 14627, USA*

¶*Center for coherence and quantum optics, Department of Physics, University of Rochester, 480 Intercampus Dr, Rochester, NY 14627, USA*

§*Materials Science, University of Rochester, Rochester, NY 14627, USA*

E-mail:

Excitation intensity dependence of diffusion lengths

Diffusion of a gas of dipolar excitons is governed by the equation, $\frac{\partial \rho(\vec{r},t)}{\partial t} = D\nabla^2\rho(\vec{r},t) + \mu I_0 \nabla(\rho(\vec{r},t)\nabla\rho(\vec{r},t)) - \frac{\rho(\vec{r},t)}{\tau_{tot}} + g(\vec{r},t)$, where $\rho(\vec{r},t)$ is the density of excitons, μ is the mobility, I_0 is the pair interaction energy and g is the generation rate (other symbols have their usual meanings from the main text).[1–3] This equation remains valid for the non-dipolar (without a permanent dipole moment) intralayer excitons considered in this work, especially at high densities.[4] However, at the low densities considered here and under the assumptions of

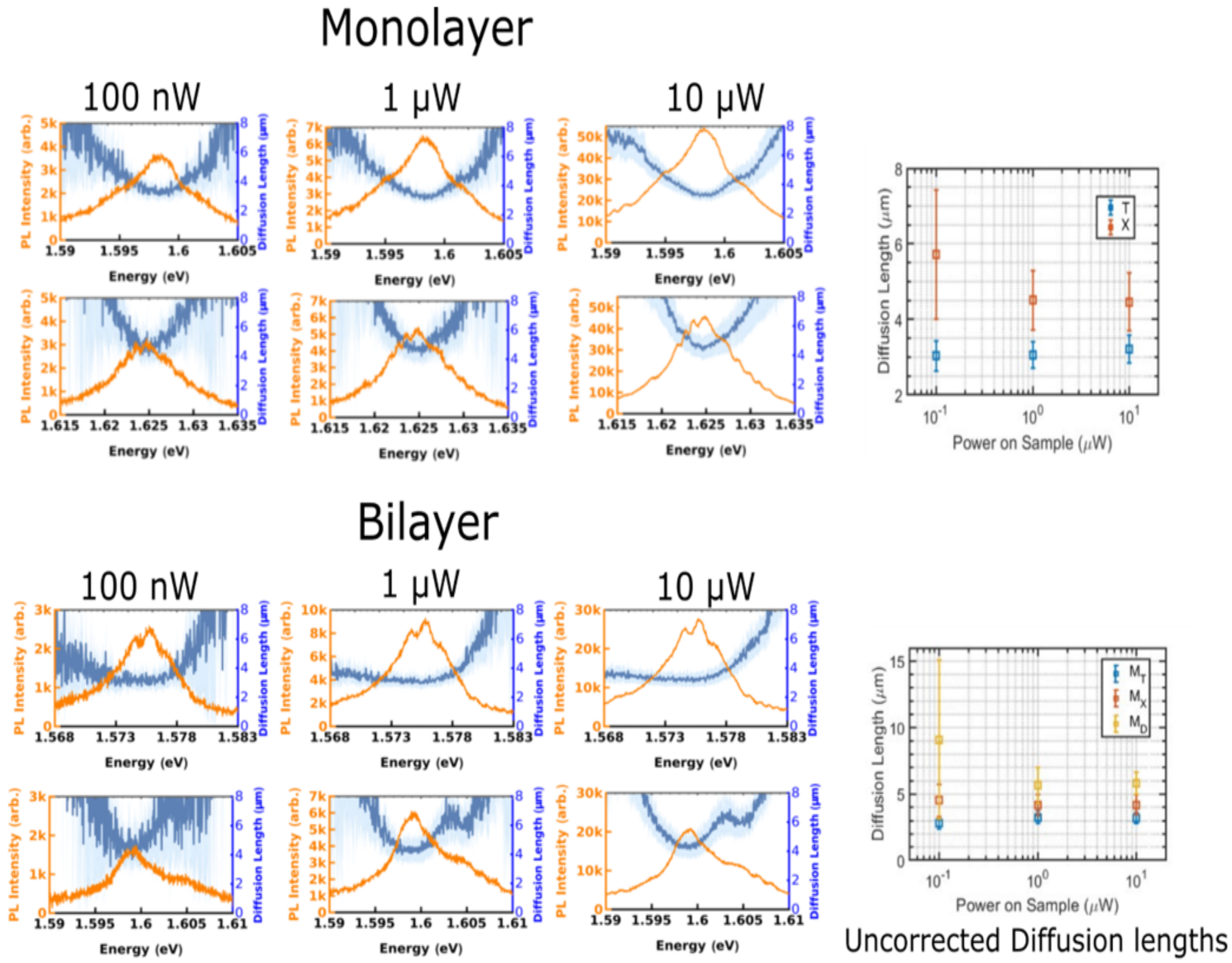

Supporting Figure 1: Diffusion lengths and spectra for the monolayer and bilayer over three order of magnitudes of excitation power, showcasing the validity of the simplifying assumptions in the paper.

steady state, the equation is simplified to retain only the effects of thermodynamic diffusion, generation, and decay to give, $D\nabla^2\rho(\vec{r},t) = \frac{\rho(\vec{r},t)}{\tau_{tot}} - \alpha I \int\int dA \exp\left(\frac{-r^2}{2\sigma^2}\right)$, where α is the absorption coefficient and I is the laser intensity, considering a gaussian profile for the laser spot. This equation can then be solved to provide the form for the steady-state profile of excitons discussed in the main text.

To substantiate the validity of the above assumptions, the uncorrected (M_X and M_D are spectrally mixed and affect each others' values of diffusion lengths) diffusion lengths are extracted over three orders of magnitudes of the excitation power in supporting figure. 1

for both the monolayer and the bilayer. The diffusion lengths are reported at the sites of quenching. As we can see at 100 nW of excitation power, the noise in the spectrum leads to large errors in the diffusion lengths for some of the species. However, despite this, we do not observe any measurable qualitative difference in the diffusion lengths for any of the species, within the bounds of error, over the ranges of excitation power considered here.

Disentangling contributions of bright and dark excitons

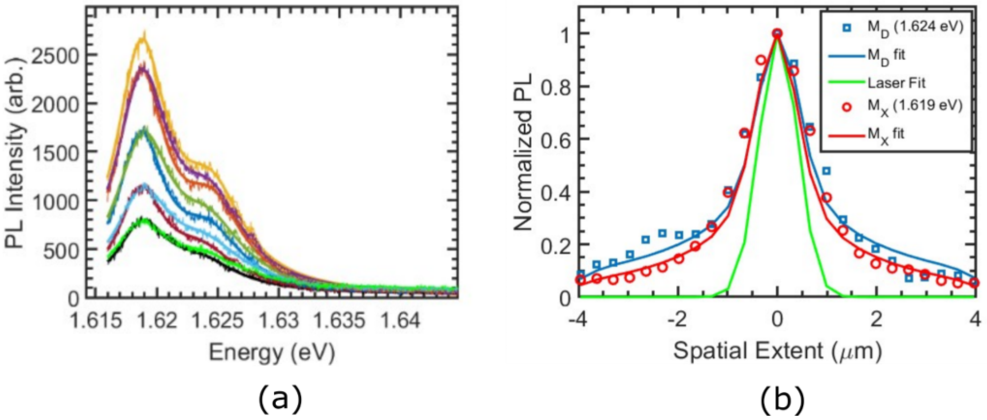

Supporting Figure 2: (a) Several spatio-spectral slices and their double-lorentzian fits, (b) Corrected spatial profile of bright and dark excitons and their respective fits, along with the fit used for the 532 nm laser spot.

In the bilayer, the spectral proximity of the bright and dark excitons causes the photoluminescence from one of the species to affect the apparent diffusion length obtained at the maximum PL intensity of the other. To remedy this we subject the spatio-spectral slices to fits of the sum of two lorentzian functions. We find that the slices lend themselves well to such a fit in supporting figure. 2 (a), which illustrates this for some representative slices. We can then subtract the contributions of the dark exciton from the spectra of the bright exciton and vice-versa. Supporting figure. 2 (b) showcases the spatial profile of the two excitons alongwith their fits as well as the gaussian fit for the laser spot used in the experiments. One of the achromatic lenses used to image the emission spot at the entrance slit of the

spectrometer-CCD setup was stepped between imaging the laser spot and the PL emission to ensure minimal defocus at both wavelengths. The small asymmetry in the diffusion of dark excitons may arise from an interplay between dark-gray exciton interconversion as well as from a more sensitive(to dielectric disorder) dipole radiation profile of the gray excitonic contribution. This fine-structure of the dark exciton is too small to be resolved in this work.[5]

Second harmonic generation measurements

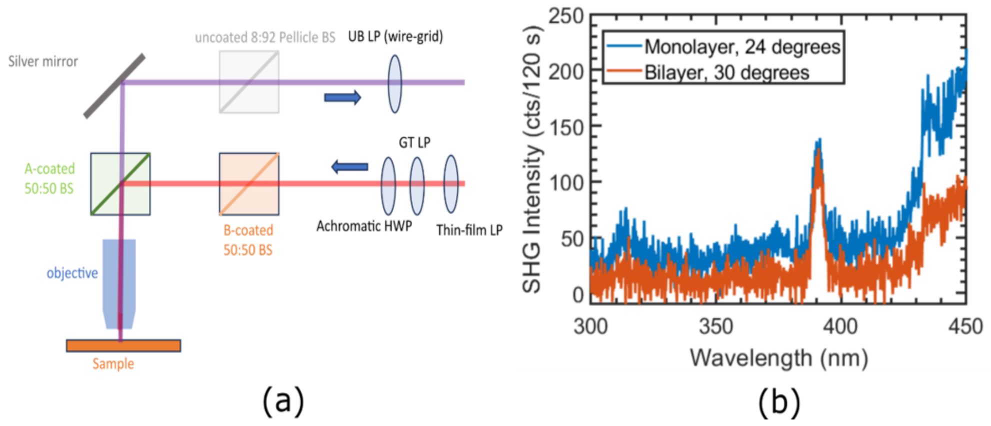

Supporting Figure 3: (a) Setup used for the SHG measurements. (b) An example of the co-polarized SHG spectra from the monolayer and the bilayer at different laser polarization angles

Two Thorlabs Elliptec piezo-rotators(ELL14) with internal coordinate references, were used synchronously to measure the co-polarized second harmonic generation signal from the monolayer and the bilayer. A Thorlabs Achromatic half-wave plate(AQWP10M-980) was used to rotate the polarization of the incident femtosecond pulsed laser at 790.5 nm. The effects of the beamsplitters in the path of the incident laser and the outgoing signal were accounted for using reflection and transmission curves obtained from Thorlabs. The effect of the beamsplitters on the linear polarization direction of either the laser or the signal was calculated to be minimal.

Temperature dependent measurements

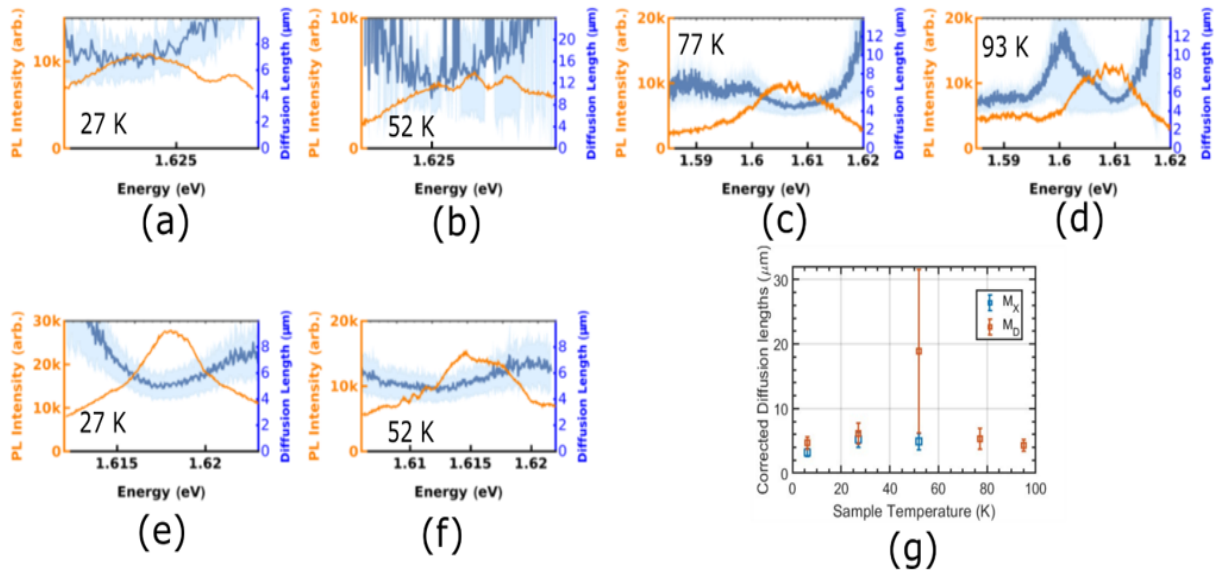

Supporting Figure 4: (a)-(d) Corrected dark exciton spectrum and diffusion lengths at the indicated temperatures, (e)-(f) corrected bright excitonic spectrum at indicated temperatures, (f) extracted diffusion lengths at maximum PL intensities. All data refers to the bilayer.

We have investigated the temperature dependence of the diffusion lengths of the bilayer in this work. We see that between 6 K and 52 K, there is a noticeable increase in the diffusion lengths of both the bright and dark excitons. This can be attributed to an increased phonon interaction at higher temperatures.[6] However, the very same mechanism is responsible for a broadening of the PL response. This together with the overall decrease in PL yield and the limitations in modelling the emission profile with lorentzian functions results in a large error in the diffusion length for the dark exciton at 52 K. Beyond 52 K, the negligible contribution from the bright excitons prevents us from obtaining an accurate estimate of the diffusion length. However, we do report a decrease in the diffusion length of the dark exciton over this range of temperature.

To establish the trionic origin of M_D^-, we consider how the ratio of the integrated PL intensities of the bright and dark trions, M_T and M_D^- evolves with temperatures. Trions being

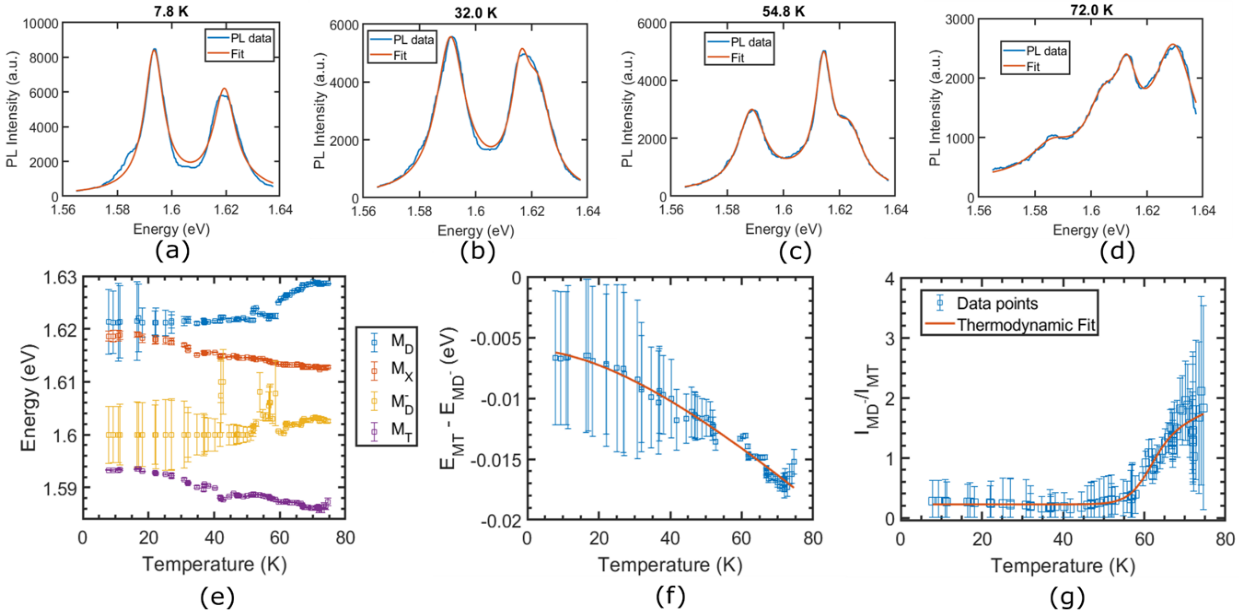

Supporting Figure 5: (a)-(d) PL spectrum and lorentzian fits to the data at indicated temperatures, (e) peak energies of the four fitted species, (f) energy difference of the bright and dark trion as a function of temperature fitted with a Varshni equation (red line), (g) ratio of integrated PL intensities and their thermodynamic fit.

fermions are assumed to obey a Fermi-Dirac step-like dependence in their optical absorption given by, $\alpha \propto \frac{1}{1+\exp\frac{T-T_0}{\gamma}}$, where values of T_0 and γ are determined by intervalley scattering processes among other things.[7] The integrated PL intensities of the species A are then given as a function of temperature by: $I_A(T) = n_A(T)\alpha_A(T)$, where $n_{M_T} = \frac{n_0}{1+\exp\frac{E(M_T)-E(M_D^-)}{k_B T}}$ and $n_{M_D^-} = \frac{n_0 \exp\frac{E(M_T)-E(M_D^-)}{k_B T}}{1+\exp\frac{E(M_T)-E(M_D^-)}{k_B T}}$, n_0 being the total density of trions in the system. The integrated PL intensities are obtained from the spectrums by fitting them with four lorentzians as shown in Supporting figure. 8 (a)-(d). The energy difference between the two species is fit with a Varshni-like equation, $E(M_T) - E(M_D^-) = \Delta_0 - \frac{\alpha_1 T^2}{T+\beta_1} + \frac{\alpha_2 T^2}{T+\beta_2}$, where α_2 is allowed to be negative to account for the blueshift of the dark trion energy with temperature. This fit of the difference in energy is used to fit the ratio of the PL intensities with their expected form in Supporting figure. 8 (f). We hence obtain the relevant values as shown in the table below.

	$T_0(K)$	$\gamma(K)$
M_T	61.18 ± 1.84	2.8417 ± 0.89
M_D	56.18 ± 1.78	2.7166 ± 0.92

PL from different spots

In the first sample, we observe the same evolution of the PL at different spots with temperature, wherever the linewidths are narrow enough for disambiguation. For spot 4, where the feature is undiscernible at 8 K, the temperature evolution still points to the population transfer.

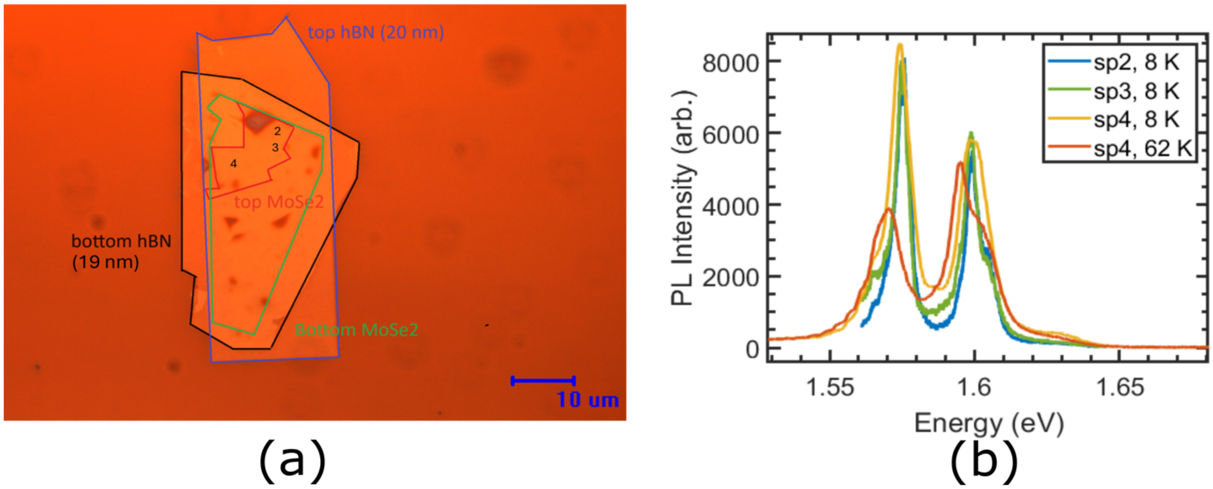

Supporting Figure 6: (a) Different spot positions within the sample, (b) PL from different spots.

AFM data of the first sample

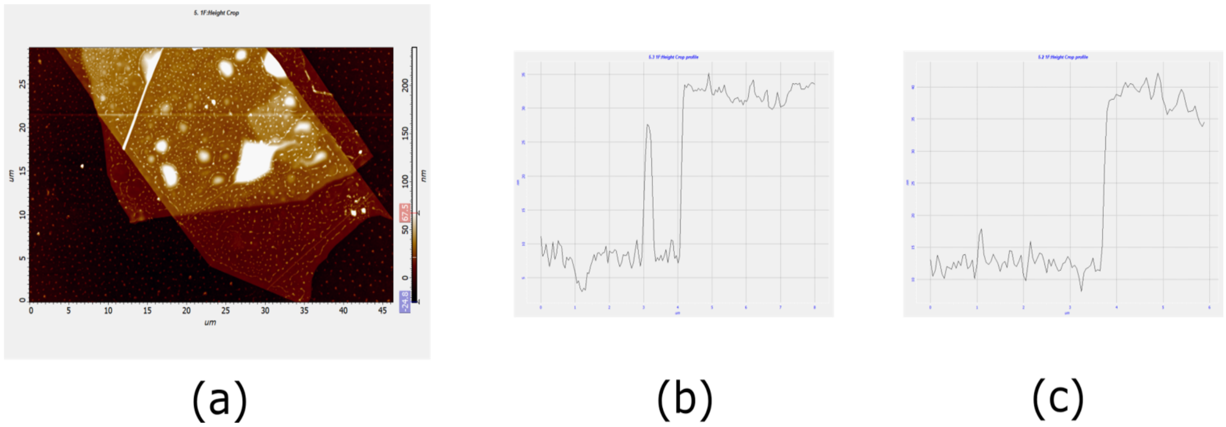

Supporting Figure 7: (a) AFM of the first sample, (b) linecut of top hBN, (c) linecut of bottom hBN

Fits of the PL spectra under a magnetic field, 532 nm excitation

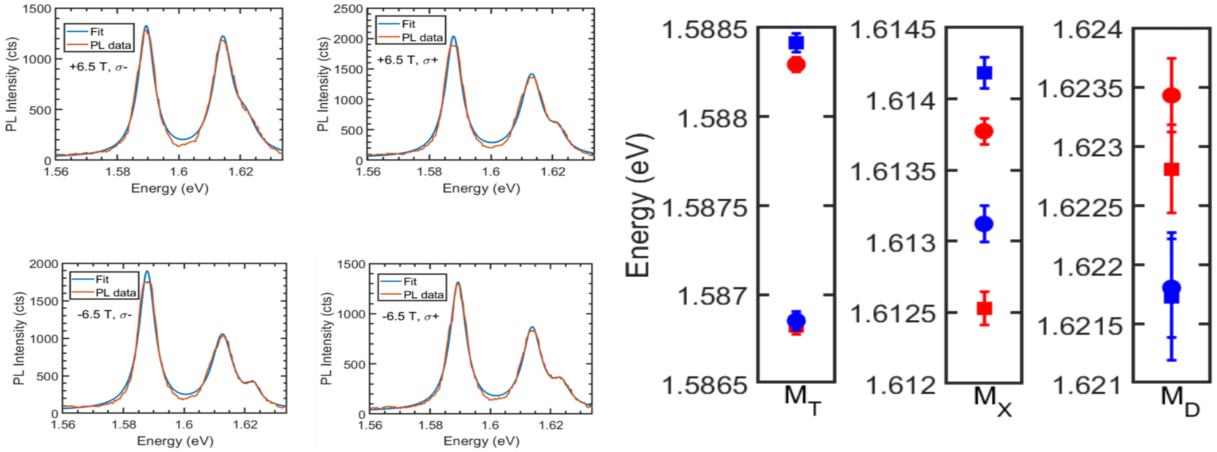

Supporting Figure 8: (left) Fits of PL spectra with three lorentzians and background, (right) extracted energies and 95 percent confidence intervals of trion, bright and dark excitons, red (blue) color represents -6.5 T (+6.5 T) while squares (circles) represents the $\sigma_-(\sigma_+)$ signal.

ChatGPT 4o transcript

In keeping with the best practices[8] in reporting the use of AI-enabled scientific communication we share the prompt and the response below:

Prompt:

This is a manuscript I have been preparing. Can you give suggestions on consistency, grammatical errors, and spelling mistakes? Please give the specific changes suggested.

Response:

Sure, here are specific suggested changes for your manuscript titled "Diffusion of brightened dark excitons in a high-angle incommensurate Moiré homobilayer":

Abstract:

Current: "twistronics, especially in low-angle twisted bilayers of transition metal dichalocogenides."
Suggested: "twistronics, especially in low-angle twisted bilayers of transition metal dichalcogenides."

Current: "While a lot remains to be known and understood about these heterostructures...
Suggested: "While much remains to be known and understood about these heterostructures..

Introduction:

Current: "These systems have been explored in a recent work22 over a range of twist angles."

Suggested: "These systems have been explored in a recent work [22] over a range of twist angles."

- Ensure consistent use of "twist angle" and "high-angle."

Results and Discussion:

Current: "We next shift our attention to the diffusion of the excitonic and trionic complexes..."

Suggested: "Next, we shift our attention to the diffusion of the excitonic and trionic complexes..."

Current: "Upon excitation with a power of 10µW in a spot of radius 1µm..."

Suggested: "Upon excitation with a power of 10 µW in a spot of radius 1 µm..."

Methods:

Current: "The monolayers and hBN (high-pressure anvil growth) were mechanically exfoliated..."

Suggested: "The monolayers and hBN (high-pressure anvil growth) were mechanically exfoliated from high-quality bulk samples obtained from 2D Semiconductors."

Current: "an achromatic lens system was used to image the emission spot..."

Suggested: "an achromatic lens system was used to image the emission spot on the CCD camera..."

Grammar and Spelling Corrections:

Current: "The diffusion lengths points us toward the presence of a more diffusive excitonic species..."
Suggested: "The diffusion lengths point us toward the presence of a more diffusive excitonic species..."

Current: "the spectral proximity of the bright and dark excitons causes the photolumines
from one of the species to affect the apparent diffusion length..."
Suggested: "the spectral proximity of the bright and dark excitons causes the photoluminescence from one of the species to affect the apparent diffusion length..."

Supplementary Information:

- **Excitation Intensity Dependence of Diffusion Lengths**:
  - **Current**: "the uncorrected (MX and MD are spectrally mixed and affect each others' values of diffusion lengths)..."
    - **Suggested**: "the uncorrected (MX and MD are spectrally mixed and affect each other's values of diffusion lengths)..."
- **Disentangling Contributions of Bright and Dark Excitons**:
  - **Current**: "achromatic lens used to image the emission spot at the entrance slit of the spectrometer-CCD setup..."
    - **Suggested**: "The achromatic lens used to image the emission spot at the entrance slit of the spectrometer-CCD setup..."
- **Temperature Dependent Measurements**:
  - **Current**: "We see that between 6K and 52 K..."
    - **Suggested**: "We see that between 6 K and 52 K..."
  - **Current**: "lorentzian functions results in a large error..."

- **Suggested**: "Lorentzian functions result in a large error..."

Consistency Checks:

- Ensure all figure citations are consistent, e.g., "Figure 2(a)" should be consistently cited as "Figure 2a."
- Verify consistent use of symbols and notation, e.g., "µm" instead of "microns" and "nm" consistently throughout.

By making these specific changes, you can improve the consistency, grammar, and clarity of your manuscript. If you need further assistance with any section, feel free to ask!

References

(1) Choi, J. et al. Moiré potential impedes interlayer exciton diffusion in van der Waals heterostructures. *Science Advances* **2020**, *6*, eaba8866.

(2) Rapaport, R.; Chen, G.; Simon, S. H. Nonlinear dynamics of a dense two-dimensional dipolar exciton gas. *Phys. Rev. B* **2006**, *73*, 033319.

(3) Ivanov, A. L. Quantum diffusion of dipole-oriented indirect excitons in coupled quantum wells. *Europhysics Letters* **2002**, *59*, 586.

(4) Sie, E. J.; Steinhoff, A.; Gies, C.; Lui, C. H.; Ma, Q.; Rösner, M.; Schönhoff, G.; Jahnke, F.; Wehling, T. O.; Lee, Y. H.; Kong, J.; Jarillo-Herrero, P.; Gedik, N. Observation of Exciton Redshift–Blueshift Crossover in Monolayer WS2. *Nano Letters* **2017**, *17*, 4210–4216.

(5) Robert, C.; Amand, T.; Cadiz, F.; Lagarde, D.; Courtade, E.; Manca, M.; Taniguchi, T.;

Watanabe, K.; Urbaszek, B.; Marie, X. Fine structure and lifetime of dark excitons in transition metal dichalcogenide monolayers. *Phys. Rev. B* **2017**, *96*, 155423.

(6) Perea-Causin, R.; Brem, S.; Malic, E. Trion-phonon interaction in atomically thin semiconductors. *Phys. Rev. B* **2022**, *106*, 115407.

(7) Arora, A.; Wessling, N. K.; Deilmann, T.; Reichenauer, T.; Steeger, P.; Kossacki, P.; Potemski, M.; Michaelis de Vasconcellos, S.; Rohlfing, M.; Bratschitsch, R. Dark trions govern the temperature-dependent optical absorption and emission of doped atomically thin semiconductors. *Phys. Rev. B* **2020**, *101*, 241413.

(8) Buriak, J. M. et al. Best Practices for Using AI When Writing Scientific Manuscripts. *ACS Nano* **2023**, *17*, 4091–4093.
